# Assessment of the effects of climate change on the occurrence of tomato invasive insect pests in Uganda

**DOI:** 10.1016/j.heliyon.2023.e13702

**Published:** 2023-02-15

**Authors:** N'dakpaze Gno-Solim Ela, Daniel Olago, Amwata Dorothy Akinyi, Henri E.Z. Tonnang

**Affiliations:** a International Centre of Insect Physiology and Ecology (*icipe*), P.O. Box 30772-00100, Nairobi, Kenya; b Institute for Climate Change and Adaptation, University of Nairobi and South Eastern Kenya University, P. O. Box 29053, Nairobi, Kenya

**Keywords:** Climate change, Invasive insect pests, Climate-pest relationship, Uganda

## Abstract

The shift in the geographical spread of invasive pests in Africa has rarely been linked directly to climate change. However, it is predicted that environmental changes play a significant role in spreading and expanding pests. The occurrence of new tomato invasive insect pests has been increasing in Uganda during the past century. Assessing the impact of temperature, rainfall, relative humidity, and windspeed on the occurrence of invasive tomato insect pests, gives a better understanding of managing and limiting the bio-invasion process sustainably. We used the Mann Kendall trend Test to establish trends in climate variables from 1981 to 2020 and to document the trend in the occurrence of new invasive pests. The relationship between climate variables and pests occurrence is analyzed using Pearson's correlation and the Generalized Linear Model (GLM-quasi-Poisson) in R-software. The results showed that temperature and windspeed have significantly increased in both Kampala and Namutumba by 0.049 °C, 0.005 m ^s−1^and by 0.037 °C, 0.003 m ^s−1^ per year respectively while in Mbale there was no change in wind speed pattern and a non-significant decrease in temperature. There was an overall rainfall increase in Kampala (*p* = 0.029) by 0.241 mm, Mbale (*p* = 0.0011) by 9.804 mm, and Namutumba (*p* = 0.394) by 0.025 mm. On the other hand, humidity has decreased both in Kampala (*p* = 0.001) by 13.3% and in Namutumba (*p* = 0.035) by 13.2% while there was a no significant change in Mbale. The results of GLM showed that each variable, taken individually, had a direct effect on the pests' occurrence in all three districts. However, with all these climate variables taken together, the effect on the pests' occurrence varied with each of the three districts; Kampala, Mbale, and Namutumba. This study demonstrated that pest occurrence is different from one agroecology to another. Our findings suggest that climate change is a driver that favors bio-invasion of tomato invasive insect pests occurrence in Uganda. It calls for awareness to policymakers and stakeholders to consider climate-smart pest management practices and policies to deal with bio-invasion.

## 1 Introduction

Food security is threatened by several factors such as environmental degradation, transboundary diseases, and insect pests [1,2]. Globally, the occurrence and proliferation of insect pests are high due to the nature of these organisms, which are poikilothermic species therefore very sensitive to climatic and environmental conditions [3]. Under global warming, the proliferation of pests has increased and the number of new invasive species discovered or reported regularly in Africa is clearly on the rise [4]. These invasive insect pests that are non-native in Africa are spreading or brought unintentionally by human activities such as international trade [1,2]. The challenge is that within the newly colonized ecozones, there are no proper natural enemies that will slow down the progression of those invasive pests [5]. Although many efforts have been made to manage the existing invasive pests and limit new introductions, climate change and climatic variability are contributing to the increase in the number of reported pests [6,7]. For example, the dry period that follows humid spells, characteristic of the climate in the Eastern part of Africa, is favorable for insect invasion and spread [8]. The report of the Intergovernmental Panel on Climate Change [9] shows an increase in the global temperature of 2 °C by 2100. Such an increase may have a drastic impact on insect pests which are essentially poikilothermic species with a high level of temperature dependency for their development and life history.

Climate change impacts result in variations in the earth's atmosphere parameters such as temperature, precipitation, wind, humidity, and soil moisture [7,10]. The changes in climate patterns increase the proliferation of pests and affect the ecosystem, including crops, animals, and human beings [7,11,12]. Therefore, climate change becomes a major threat to smallholder farmers making them more vulnerable to pests invasion and new diseases [13].

As a result of these variabilities, there have been a plethora of invasive pests in sub-Saharan Africa (SSA) in the last decade. In Uganda, agriculture plays an essential role in the country's economy. Mbale and Namutumba are among the districts where most of the tomatoes produced in Uganda are grown [14,15]. Tomato production is the primary source of income for smallholder farmers in these districts. Farmers have been affected by several insect pests causing huge yield losses in the past three decades. Besides, the country has witnessed rainfall fluctuations, strong winds, rising temperatures, floods, and drought occurrences, especially in the recorded pests' locations [16]. Also, changes in moisture content hasten the development of insects while changing the interaction between the pest, their natural enemies, and their hosts [1]. Moreover, pest response to increasing temperatures has an impact on the amount of damage to crop yields [17]. The highest proliferation of some insect pests occurs at the highest temperatures and lowest relative humidity [18,19].

Establishing the relationship between the biology of the pests and the relative humidity is crucial to understanding the fast invasion of a pest in certain regions [20]. However, there is limited scientific evidence that demonstrates the rate of establishment of invasive pests in Uganda as a result of recent climate change. To better understand the establishment of new pests and to develop more effective early warning and control approaches, it is key to comprehend the relationship between bio-invasion and environmental change. Therefore, our research aims to investigate the effect of environmental changes in the districts by assessing the trends in temperature, rainfall, relative humidity, and windspeed and their effects on the rate of the occurrence of tomato invasive pests.

## 2 Methodology

### 2.1 Study area

**Fig. 1:**
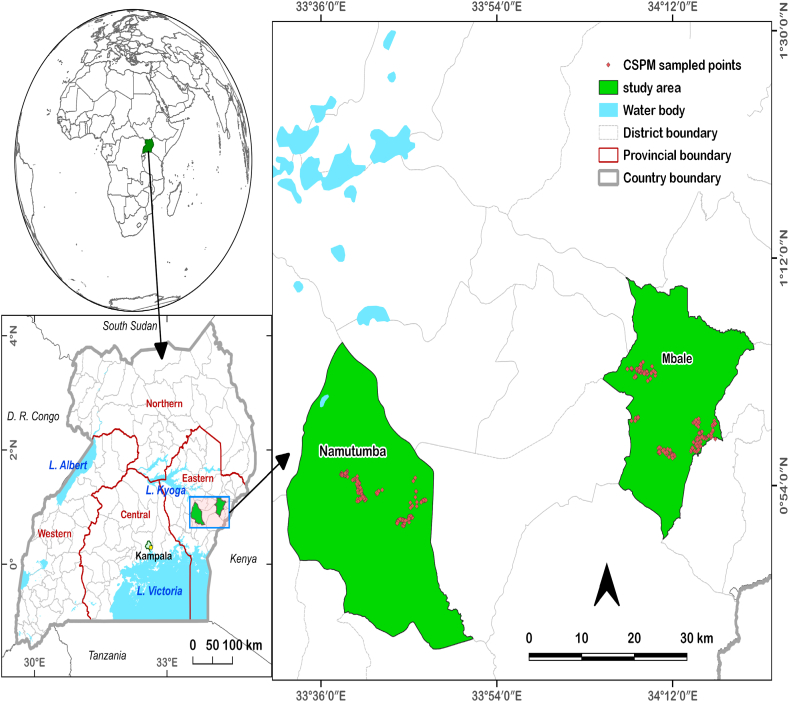
Location of Uganda in Africa, author.

The study was conducted in Uganda. Uganda is located in East Africa (Fig. 1) and is a landlocked country bordered to the North by Sudan, Kenya to the west, to the South by Lake Victoria, Rwanda, Tanzania, and the Democratic Republic of Congo [21]. Uganda lies between latitude 0.5° N and longitude 32.0° E with an elevation of approximately 1137 m above sea level [22]. Most of the smallholder farmers in the study area rely exclusively on rainfall for their agriculture. The common crops grown in the area are maize, beans, cassava, banana, and tomato. For many years, the farmers have been struggling with the invasion of their croplands by pests that cause between 10 and 100% crop losses. The most common ways used in the study area to deal with the pests are the use of herbicides, pesticides, fungicides, and insecticides. Though we used Kampala as the reference point, two regions of tomato cultivation areas were assessed to compare the impact within those regions. The two regions were Mbale (34.181°E, 1.0784°N) and Namutumba (33.6861°E, 0.8361°N). The coordinates represent the centroids of the districts.

### 2.2 Data collection

The climate variables used in the study were temperature, rainfall, windspeed, and relative humidity obtained from the years 1981–2020 to capture changes in the climate observed within the forty years [23,24]. These data were retrieved from NASA power (https://power.larc.nasa.gov/data-access-viewer/) at a spatial resolution of 0.5 × 0.625° latitude/longitude. The annual averages of these bioclimatic variables were used to match the number of invasive pests recorded annually [4]. The data on the invasive insect pests were obtained from online databases such as the European and Mediterranean Plant Protection Organization (EPPO) available at https://www.eppo.int/, the Global Biodiversity Information Facility (GBIF) (https://www.gbif.org/), and the Centre for Agriculture and Bioscience International (CABI) (https://www.cabi.org/) [25,26]. All these are huge online species occurrence databases that house billions of records collected globally since the 1900s [27]. The native species that were endemic to Uganda were excluded from the analysis. These were identified from the CABI (n = 4) databases as they are precisely categorized as native Ugandan species and would thus not be ideal as invasive for this current study. Elimination of native species has also been conducted in similar studies such as by Huang et al. [4]. The total number of species that were finally used in this analysis after elimination of the native species from the data obtained from the different databases were thirty eight. To standardize the data, the cumulation was estimated from year to year. From the European and Mediterranean Plant Protection Organization, no reported insect pest species have entirely disappeared from Uganda, and approximately 80% of the recorded tomato invasive insect pest's location was in Kampala (32.59^o^ E, 0.32^o^ N). This was expected as the Kampala agricultural produce markets are central to all the agricultural products originating across the country hence many pests also become introduced and centralized in Kampala. Additionally, this could be attributable to the fact that the capacity to identify, record, and report these pests could be higher in Kampala than in other parts of the country.

### 2.3 Data analysis

#### 2.3.1 Trend analysis

The Mann-Kendall (MK) trend method, which is a non-parametric test, was used on the climatic variables to assess the negative or positive tendency in the level of temperature, rainfall, relative humidity, and windspeed over the years [[28], [29], [30]]. The MK analysis is commonly used for environmental and climate data to understand the significance level of the tendency in time series data within a location and to establish the occurrence of climatic changes [31,32]. Specifically, the analyzed data provided insight into whether there is a significant increase or decrease trend in the historical time series of the chosen variables in our case the temperature, rainfall, relative humidity, and windspeed. The trends can be negative, non-null, or positive. When there is a positive value of the MK test, there is an increasing trend while the opposite shows a decreasing trend [33,34].

The statistical equation (1) of the MK test is given:

1 $$\sum_{a=1}^{n-1}\sum_{b=a+1}^{n}sign=\left(x_{b}-x_{a}\right)\;\left(SEQ\;Equation\;\backslash *\;ARABIC\;1\right)$$

With:

*x*_*b*_ = the value of the climatic variable (temperature, rainfall, relative humidity, or windspeed) data at time b (second time step e.g., 1982)

*x*_*a*_ = the value of the climatic variable (temperature, rainfall, relative humidity, or windspeed) data at time a (initial time e.g., 1981)

where equation (2) is:

2 $$sign\;\left(x_{b}-x_{a}\right)=\left\{ \begin{array}{c} 1,if\;\left(x_{b}-x_{a}\right)>0\\ 0,if\;\left(x_{b}-x_{a}\right)=0\\ -1,if\left(x_{b}-x_{a}\right)<0 \end{array}\right.\left(SEQ\;Equation\;\backslash *\;ARABIC\;2\right)$$

The Sen's slope estimator test is used to determine the statistical significance of the tendency in climatic variables over time [35,36]. For a set of pairs (a, x_a_) the Sen's slope equation (3) is:

3 $$\frac{x_{b}-x_{a}}{b-a}\left(SEQ\;Equation\;\backslash *\;ARABIC\;3\right)$$

#### 2.3.2 Statistical analyses

The effect of climate variables on pest invasion over the years was assessed using a Generalized Linear Model (GLM) because the data was ‘count data’ [37,38]. This model was used to predict pest occurrence and was well suited for our count data [39]. The Shapiro test was first considered to test the normality of the count. Thereafter, the GLM was run using the entire dataset occurrence and in R-software using the package ‘quasipoisson’ and used to determine the effects of the climate variables i.e., temperature, rainfall, relative humidity, or windspeed on the occurrence of invasive insects' pests [40]. Equation (4) was used to perceive the impact of the changes in the climatic variables over the years on the occurrence of new pests in Uganda. Quasi-Poisson models are better when dispersion is not close to one as assumed by the Poisson model. Equation (4) is as stipulated:

4 $$E\left(Y\right)=\mu =\exp(\beta_{0}+\beta_{1}x_{1}+\beta_{2}x_{2}+....+\beta_{n}x_{n})$$

where.

- βs represents the expected change in the **log of the mean** per unit change in *X.*
- exp(β) is the effect of the independent variable on the mean.

The Quasi-Poisson GLM that was run in R software is: glm(formula = Count ∼ the value of the climatic variable (temperature, rainfall, relative humidity, or windspeed) data, family = quasipoisson(link = log)).

Furthermore, Pearson's correlation analysis was used to determine the association between the tomato invasive insect pest occurrence and climate variables.

## 3 Results

### 3.1 Assessment of the occurrence of the tomato new insect pests

**Fig. 2:**
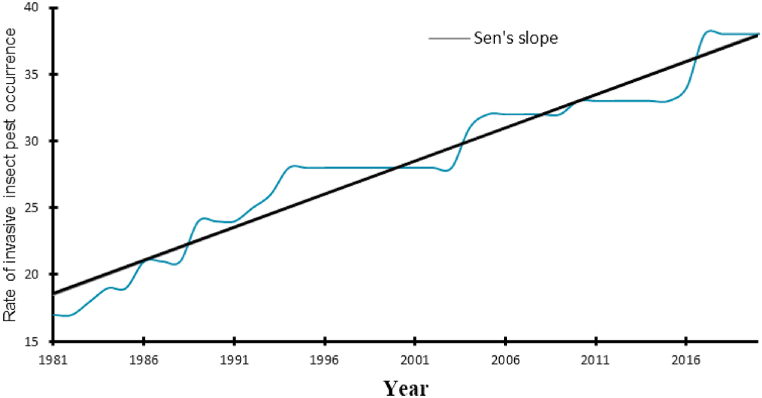
Occurrence of tomato new insect pests over the 40 years (1981–2020) in Uganda.

The MK test showed an increase in the occurrence of tomato new pests over the years (*n* = 38) (Fig. 2).

### 3.2 Estimation of temperature trends

**Fig. 3:**
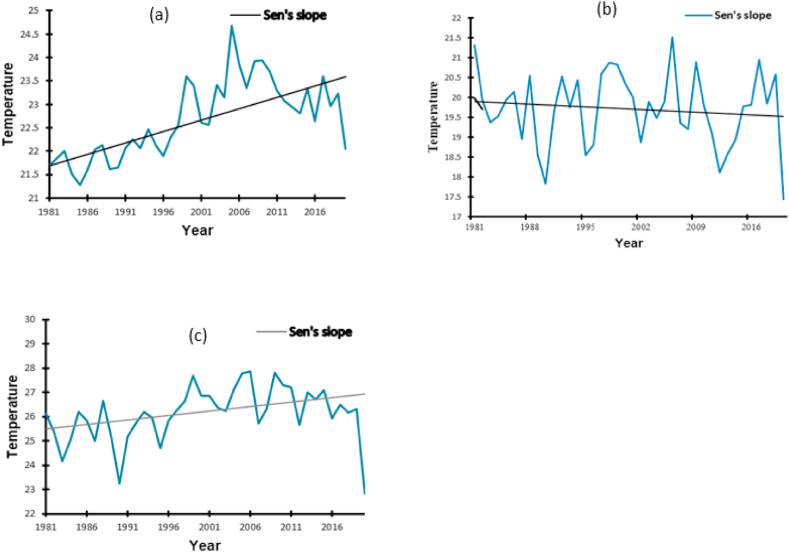
The annual temperature trends from 1981 to 2020 in Kampala (a), Mbale (b) and Namutumba (c).

The MK and Sen's slope trends for the annual temperature for the three districts are presented in Table 1 and Fig. 3, respectively. There was a significant increase in Kampala (*p* < 0.0001) and Namutumba (*p* = 0.006) by 0.049 °C and 0.037 °C per year, respectively. Over the years, we observed little decrease (0.002 °C) with no significance in the trend variation of mean temperature in Mbale (*p* = 0.894) (Table 1). The highest increase in temperature occurred in Namutumba between 1981 and 2020 and reached a maximum of 27.88 °C (Table 1).

**Table 1:** Estimated Sen's slope values for the temperature variable trends from 1981 to 2020 in the districts.

Location	Range		Sen's slope	*p*-Value
	Minimum	Maximum		
**Kampala**	21.28	24.68	0.049	<0.0001
**Mbale**	17.43	21.51	−0.002	0.894
**Namutumba**	22.84	27.88	0.037	0.006

### 3.3 Determination of rainfall trends

**Fig. 4:**
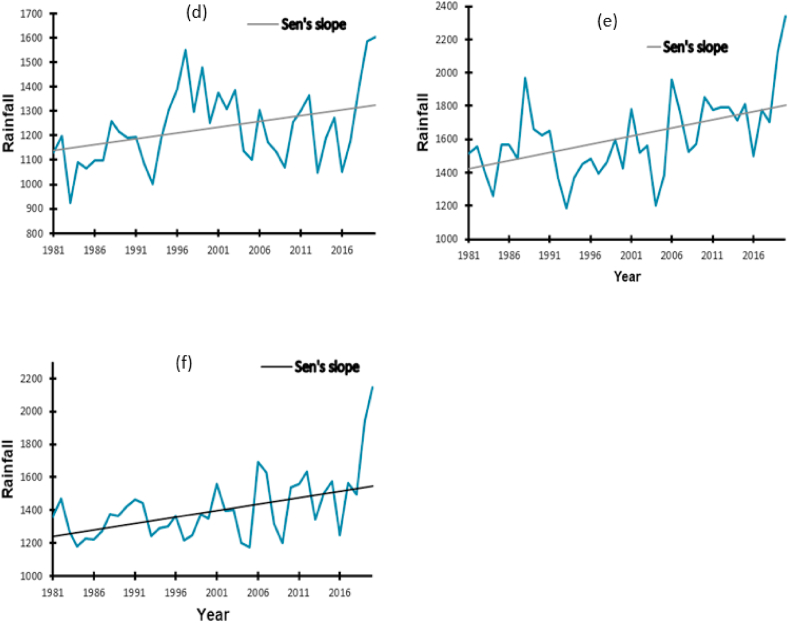
Annual rainfall trends from 1981 to 2020 in Kampala (d), Mbale (e), Namutumba (f).

The MK and Sen's slope trends for the annual rainfall for the three districts are presented in Table 2 and Fig. 4, respectively. There was a significant increment in rainfall in Kampala (*p* = 0.029) and Mbale (*p* = 0.0011) by 0.241 mm and 9.804 mm per year, respectively.

**Table 2:** Estimated Sen's slope values for the rainfall variable trends from 1981 to 2020.

Location	Range		Sen's slope	*p*-value
	Minimum	Maximum		
**Kampala**	923.92	1602.92	0.241	0.029
**Mbale**	1187.88	2341.03	9.804	0.0011
**Namutumba**	1174.05	2144.79	0.025	0.394

There was no significant difference in rainfall trends in Namutumba (*p* = 0.394). However, there was an increase in rainfall by 0.025 mm per year. Overall, there was an increase in rainfall amounts in the three districts (Table 2).

### 3.4 Estimation of windspeed trends

**Fig. 5:**
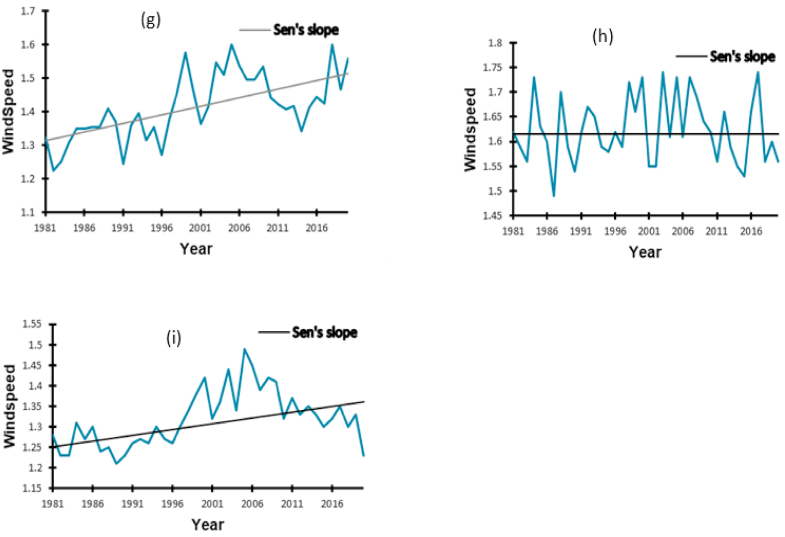
Annual windspeed trends from 1981 to 2020 in Kampala (g), Mbale (h), Namutumba (i).

There is a significant variation in the windspeed over the 40 years in Kampala (*p* < 0.0001) and Namutumba (*p* = 0.002) with increasing speed respectively, as little as 0.005 m ^s−1^ and by 0.003 m ^s−1^ per year (Table 3, Fig. 5). Higher windspeed increases insect movement. However, no significant variation in windspeed was reported in Mbale (Table 3).

**Table 3:** Estimated Sen's slope values for the windspeed variable trends from 1981 to 2020.

Location	Range		Sen's slope	*p*-value
	Minimum	Maximum		
**Kampala**	1.23	1.60	0.005	<0.0001
**Mbale**	1.49	1.74	0	0.953
**Namutumba**	1.21	1.49	0.003	0.002

### 3.5 Determination of humidity trends

**Fig. 6:**
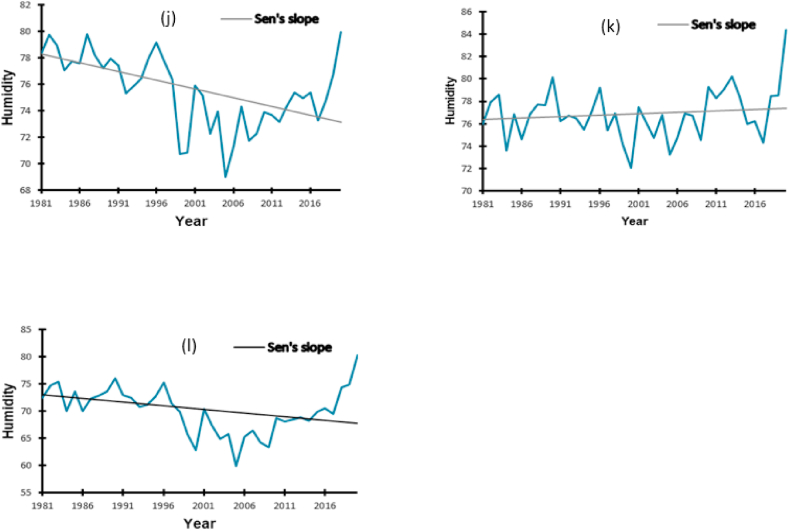
Annual relative humidity trends from 1981 to 2020 in Kampala (j), Mbale (k), Namutumba (l).

The last climate variable considered to affect insect pest occurrence is humidity. The abnormalities in the annual relative humidity trends are clear evidence of an unpredictable moisture pattern in the three districts. The MK and Sen's slope test showed a trend in the time series data with a significant decrease in humidity over the years by 13.3% RH in Kampala (*p* = 0.001) and by 13.2% RH in Namutumba (*p* = 0.035). In Mbale, there was no significant difference in the humidity trend (*p* = 0.395). In Mbale, there was no significant difference in the humidity trend (*p* = 0.395). All these trends are captured in Table 4 and in Fig. 6.

**Table 4:** Estimated Sen's slope values for the relative humidity trends from 1981 to 2020.

Location	Range		Sen's Slope	*p*-value
	Minimum	Maximum		
**Kampala**	69.03	79.91	−0.133	0.001
**Mbale**	72.06	84.38	0.025	0.395
**Namutumba**	59.88	80.31	−0.132	0.035

### 3.6 Assessments of the relationships between climate variables and the occurrence of new insect pests in kampala, mbale, and namutumba districts

**Fig. 7:**
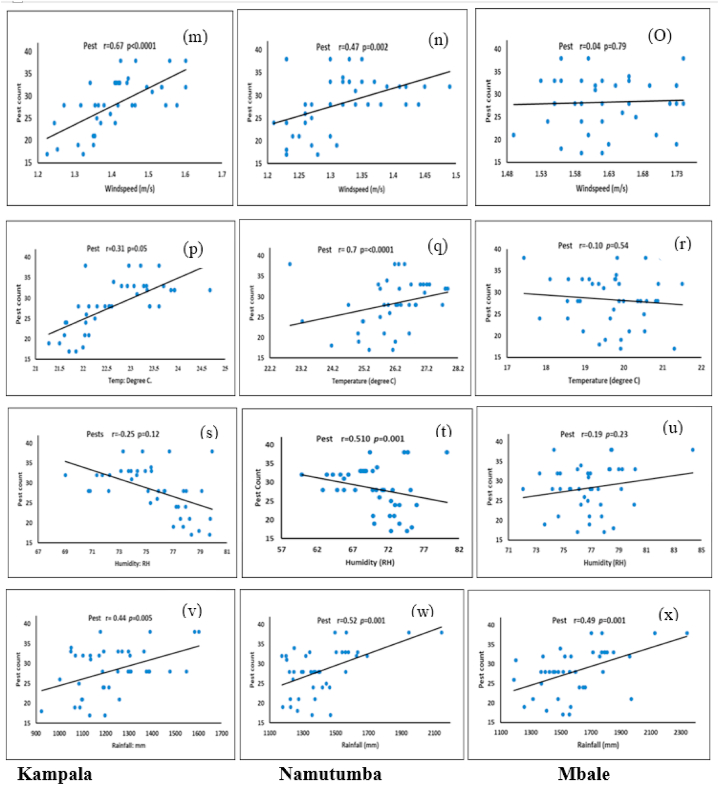
Associations assessed by a Pearson's correlation analysis, among windspeed, temperature, humidity, rainfall and occurrence of tomato invasive insect pests (pest count) in Kampala [(m); (p); (s); (v)], in Namutumba [(n); (q); (t); (w)], in Mbale [(O); (r); (u); (x)]; **r** is correlation coefficient.

The results of the relationships among the occurrence of invasive tomato insect pests, windspeed, temperature, humidity, and rainfall assessed by Pearson's correlation, are presented in Fig. 7. There were negative relationships between the humidity variable and pest occurrence in Kampala and Namutumba as well as temperature variable and pest occurrence in Mbale. The relationships among windspeed, temperature, rainfall variables, and pest occurrence were positive in Kampala and Namutumba. The positive relationships observed in Mbale were among windspeed, humidity and rainfall, and pest occurrence. The associations among all studied climate variables and pest occurrence were significantly correlated (*p* < 0.05) in the three districts, except for humidity in Kampala, windspeed, temperature, and humidity in Mbale.

The results of the GLM analysis estimating the effect on pest occurrence of each climate variable are shown below (Table 5). Due to overdispersion with the Poisson, we used the quasi-Poisson model. The GLM was fitted to the observed rainfall, temperature, humidity, and windspeed in the occurrence of invasive insect pests in Kampala. Air temperature (χ^2^ = 31.331, degrees of freedom (df) = 1, *p* = 2.176^e−08^), relative humidity (χ^2^ = 12.23, df = 1, *p* = 0.0005), rainfall (χ^2^ = 8.4699, df = 1, *p* = 0.004) and wind component (χ^2^ = 28.868, df = 1, *p* = 7.749^e−08^) at various pressure levels were all significant in the occurrence model. Rainfall, temperature, and windspeed affected positively pest occurrence counts while relative humidity affected the occurrence negatively. Even though in an agroecology, the climate variables influence pest occurrence, windspeed (*p* < 2.2^e−16^) is much more significant than rainfall (*p* = 8.310^e−12^) and temperature (*p* = 6.144^e−08^). The variations simultaneously in both windspeed and temperature are the major effect in the pest occurrence (*p* = 6.102^e−11^). The concurrent variations in all the variables are likely to have a low effect on the pest occurrence (*p* = 0.054) (Table 5).

**Table 5:** Generalized linear model (GLM) terms for estimating pest count, as a function of climate variables, of the three districts: Kampala, Mbale, Namutumba.

Dis.		Rainfall	Temperature	Humidity	Windsp.
Kam	LR Chisq	8.4699	31.331	12.23	28.868
	Df	1	1	1	1
	Pr(>Chisq)	0.004	2.176e^−08^	0.0005	7.749e-08
Mba	LR Chisq	11.877	0.38599	1.4828	0.074178
	Df	1	1	1	1
	Pr(>Chisq)	0.0007	0.5344	0.23	0.7853
Nam	LR Chisq	12.893	3.8541	2.4605	9.7638
	Df	1	1	1	1
	Pr(>Chisq)	0.0003	0.04962	0.1167	0.00178

Significance: ***p < 0.001, **p < 0.01 *p < 0.05. Relative humidity: Rh; windspeed: Ws; rainfall: Rf; temperature: T.

The significance of climate variables in Mbale on the occurrence of pest counts varied in the model (Table 5). Significant differences in occurrence were only observed in rainfall (χ^2^ = 11.877, df = 1, *p* = 0.0007). On the contrary there was no significant influence of temperature (χ^2^ = 0.38599, df = 1, *p* = 0.5344) windspeed (χ^2^ = 0.074178, df = 1, *p* = 0.78) and relative humidity (χ^2^ = 1.4828, df = 1, *p* = 0.23) on the invasive insect pests counts. In this case, rainfall is the only parameter that (*p* < 2.2^e−16^) affects the invasive insect pest occurrence when the variables are combined in one model. The variations in relative humidity, windspeed, and rainfall (*p* = 0.028) as well as the variations in relative humidity, windspeed, and temperature (*p* = 0.027) are the only cases of significant change in the pest occurrence.

The effects of climatic variables on invasive insect pests in Namutumba have similar results in Kampala. Their shared trends could explain this similarity and effect (Table 5). Similarly to Kampala, rainfall in Namatumba had a significant effect on the pest (χ^2^ = 12.893, df = 1, *p* = 0.0003). Among other variables, temperature did influence pest occurrence (χ^2^ = 3.8541, df = 1, *p* = 0.04962) as well as windspeed (χ^2^ = 9.7638, df = 1, *p* = 0.00178) compare to relative humidity which had no significant influence pest occurrence (χ^2^ = 2.4605, df = 1, *p* = 0.1167). The new occurrence of invasive insect pests in Namutumba agroecology where all the climate variables interact together is highly influenced respectively by windspeed (*p* = 7.612^e−06^), rainfall (*p* = 0.002), humidity (*p* = 0.04) (Table 5). Similarly, the interaction between windspeed and rainfall significantly affects the probability of new pest invasion (*p* = 0.021).

## 4 Discussion

In this study, we provided information on climate trends and their effect on insect pest occurrence in Uganda specifically the cultivated tomato areas in Kampala, Mbale, and Namutumba. Our results have shown an increasing annual temperature trend in Kampala and Namutumba over the last forty years by 0.04 °C annually.

Our study showed that the rainfall trends increased significantly in Kampala (0.24 mm) and Mbale (0.0011 mm), with a significant decrease in the humidity in Kampala and Namutumba confirming the findings of Ssentongo et al*.* [41], who reported, an overall decrease in the humidity in Uganda of about 12% during the past 34 years in Uganda. This variation could have been introduced by the differences in the sources of data.

The trends of windspeed amplified between 1981 and 2020, our data shows an increased rate of 0.05 m ^s−1^ and 0.003 m ^s−1,^ respectively, in Kampala and Namutumba during the study period. The rise of invasive insect pests in Uganda is wind-aided due to the positive relationship between the wind and pest data. This validates previous assumptions that the migration of some pests is favored by strong winds, which propel them [[42], [43], [44]].

The variations in the annual precipitation trends are evidence of unpredictable rainfall patterns in the study area. Therefore, it is evident that rainfall has a significant positive relationship with pests [45]. The increase in rainfall in these historical data indicated moments of flood seasons. Additionally, the analysis of rainfall variability in Uganda has proven that extreme events such as floods and mainly droughts have distorted tomato productivity through diseases and insect pests, calling for adaptive measures to help farmers mitigate these challenges [46]. A study by Khan [47] showed that the population of leafhopper and Jassid was at their highest when relative humidity was reduced, which confirms the negative correlation between Rh and insect pest incidence in Kampala and Namutumba. At the same time, *Chilo partelous* propagates faster when relative humidity increases which might be the case of our study in Mbale where there is positive correlation between Rh and pest occurrence [18]. This study established the general trend, however, further studies may address the specific species that decrease or increase in the variation of the climatic variables.

Overall, the rate of pest occurrence trend increased across the three districts. The changes in the temperature and windspeed patterns are associated with the rise of pests. This suggests that there is a meaningful trend in the association between increasing pests and climate variables. Our findings are similar to those reported by Haack et al*.* [4] who found a positive impact of temperature on the increase of the establishment rate of invasive alien insects in China, the United Kingdom, and the United States. Likewise, our results agree with previous results from a study by Phophi et al. [48] who concluded that climate change was the most important factor that influenced pests outbreaks and invasion in the Limpopo province of South Africa [48]. Scientific studies jointly done by the International Centre of Insect Physiology and Ecology (*icipe*) and the Centre for Agriculture and Bioscience International (CABI) have reported that climate change was a major driver of transboundary pests’ establishment in Eastern Africa. Besides, rainfall and temperature variability are susceptible to influencing pests occurrence thereby affecting tomato production [45,49]. Invasive pests such as leaf miner and thrips influence tomato yield loss and quality [[50], [51], [52]]. While the climatic conditions seemed favorable, the breakout of insect pests could have negatively impacted tomato production. Moreover, the GLM with quasi-Poisson results showed a significant impact of climate variables on pests. Even though the causes of bio-invasions are multifaceted, changes in abiotic and biotic components of the environment were recognized as primary drivers of species invasion [53]. The rest of the variations in pests that the model could not capture as a function of the climatic variables could be explained due to other factors, such as trade and exchange of cultivars. Overall, extreme temperatures, changes in rainfall, and windspeed patterns increase the risk of invasive insect species introduction with an expansion of their geographic range [2].

Finally, the present study has utilized secondary data and encourages its re-use for addressing further research questions., Our study suggests that the increase in establishment rates of invasive insect pests in Uganda, during the past four decades can be partially explained by climate change given that temperature, rainfall, wind speed, humidity can facilitate bio-invasion.

## 5 Conclusion

This study examined the relationship between climate change and variability factors and invasive insect pests in Uganda. The study focused mainly on temperature, rainfall, relative humidity, and windspeed. The analysis was done using the MK trend and Sen's slope test to observe the trends in the climate variables over 39 years from 1981 to 2020. The results showed some significant increase in the temperature in Kampala, Namutumba and no significant variation in Mbale, a significant increase in windspeed in Kampala and Namutumba with no variation in Mbale. Also an increase in rainfall in the three districts (more significant in Mbale) while a decrease of humidity (no significant in Mbale). This is a clear indication that climate change has occurred in Kampala, Namutumba, and Mbale. The GLM analysis concluded that variation in both windspeed and temperature is the major effect in the pest occurrence in Kampala. For Mbale, simultaneous changes in humidity, windspeed, rainfall as well humidity, windspeed, and temperature caused a significant occurrence of invasive insect pests while for Namutumba, it is the interaction between windspeed and rainfall. It showed that changes in climatic variables impacted differently the proliferation and occurrence of tomato invasive insect pests in each district. This study confirms that the expected occurrence curve can increase in the changing conditions. So, changes in temperature, windspeed, and humidity climate patterns are more conducive to the survival and occurrence of the invasive insect pest in Kampala and Namutumba than in Mbale, which is another stress to crop production.

## 6 Limitations

Because of the lack of information about the exact date of bio-invasion of a pest in Mbale and Namatumba, we assume the dates of recordings of pests' introduction in the regions are taken to be the same date the pests invade Mbale and Namutumba. Another limitation of this study is the nature of data, as mentioned above, we used secondary data which may contain noises that reduce the accuracy of the analysis.

## Declarations

### Author contribution statement

N'dakpaze Gno-Solim Ela: Conceived and designed the experiments; Performed the experiments; Analyzed and interpreted the data; Wrote the paper.Daniel Olago: Conceived and designed the experiments; Contributed reagents, materials, analysis tools or data.Amwata Dorothy Akinyi: Conceived and designed the experiments; Analyzed and interpreted the data; wrote the paper.Henri E. Z. Tonnang: Contributed reagents, materials, analysis tools or data; Analyzed and interpreted the data; Wrote the paper.

### Funding statement

This work was supported by the German 10.13039/501100006456Federal Ministry for Economic Cooperation and Development (10.13039/501100006456BMZ) commissioned and administered through the Deutsche Gesellschaft fürInternationaleZusammenarbeit (10.13039/501100011099GIZ) Fund for.International Agricultural Research (10.13039/501100015633FIA), grant number 18.7860.2–001.00.

### Data availability statement

Data are available upon request to the corresponding author.

## Declaration of competing interest

The authors declare that they have no known competing financial interests or personal relationships that could have appeared to influence the work reported in this paper.
